# Acquired perforating dermatosis with associated complicated cellulitis and amputation in a hemodialysis patient 

**DOI:** 10.5414/CNCS110297

**Published:** 2021-03-11

**Authors:** Ana Domingos, Roberto Calças, Eduarda Carias, Joana Vidinha, Anabela Malho Guedes, Viriato Santos, Patrick Agostini, Francisco Ildefonso Mendonça, Pedro Leão Neves

**Affiliations:** 1 Division of Nephrology, Centro Hospitalar e Universitário do Algarve; 2 Pathological Anatomy Laboratory, Centro Hospitalar Universitário do Algarve – Faro-Portimão; 3 Division of Dermatology, Centro Hospitalar e Universitário do Algarve, Faro, and; 4 University of Algarve – Department of Biomedical Sciences and Medicine, Faro, Portugal; *Joint authorship

**Keywords:** acquired perforating dermatosis, cellulitis, Serratia, diabetes, hemodialysis

## Abstract

Introduction: Cutaneous manifestations related to chronic kidney disease (CKD) are common and associated with high morbidity. Acquired perforating dermatosis (APD) occurs mostly in diabetic or CKD patients, namely those undergoing hemodialysis. Case report: A 58-year-old male with type 2 diabetes, with long-term insulin use, several microvascular and macrovascular complications, and on maintenance hemodialysis for 5 years presented with a 1-week history of painful, pruritic, umbilicated papules and some punctiform necrotic lesions on his left forearm, both hands, and both amputation stumps. There was no evidence of infection or vascular alterations, and the patient was not responsive to an initial course of topical corticosteroid. These lesions rapidly evolved to larger, coalescent necrotic injuries, with aggravated pain, intense left-hand skin peeling, and the appearance of similar lesions in the trunk, requiring hospital admission. Calciphylaxis and APD were suspected. Skin biopsy was performed and directed treatment initiated, including intradialytic sodium thiosulfate. Histology findings were compatible with APD and also excluded findings suggestive of vasculitis or calciphylaxis. Soon after, difficult-to-treat cellulitis of the left hand and forearm progressed to critical ischemia and amputation. Microbiology analysis revealed *Serratia marcescens* as the causative agent. Discussion: To our knowledge, there are no previously described cases of APD-related cellulitis. Its treatment can be particularly challenging, as lesions can persist and relapse, and chronic scars can develop. *S. marcescens* behaves as an opportunistic and difficult-to-treat pathogen, complicating the prognosis. Conclusion: APD can be associated with cellulitis and all of its complications in patients with underlying severe vasculopathy. Awareness of this complication in APD with early referral and aggressive treatment might improve the outcomes and quality of life of such patients.

## Introduction 

Perforating dermatosis comprises a group of conditions in which transepidermal elimination of material from the upper dermis occurs (including collagen, elastic fibers, necrotic connective tissue, and keratin, among others) [1, 2, 3, 4]. Acquired perforating dermatosis (APD), a term introduced in 1989 by Rapini et al. [5], encompasses Kyrle’s disease, perforating folliculitis, acquired elastosis perforans serpenginosa, and acquired reactive perforating collagenosis, which were previously thought to be separate disorders. APD occurs in adult patients and is most frequently found in patients with diabetes or chronic kidney disease (CKD), particularly among those undergoing hemodialysis [1, 2, 10], in whom the prevalence of APD can reach 11% [3, 4, 6]. APD is of particular concern when the cause of renal failure is diabetic nephropathy [2]. Other associated pathologies have been identified, such as human immunodeficiency virus (HIV), tuberculosis, neoplasia, liver disease, hypothyroidism, or hyperparathyroidism [1, 3, 8]. APD has no geographical or racial association [7]. Both sexes seem to be equally susceptible, although some authors describe male predominance [8]. The average age of APD presentation is 56 years [6]; however, the pathogenesis of APD remains unclear [2, 4, 8]. 

To our knowledge, there are no previously described cases of APD-related cellulitis. Awareness of this complication coupled with early referral and aggressive treatment might improve patient outcomes, whereas concomitant vasculopathy seems to significantly worsen the prognosis. Here, we present a case report and brief literature review of a case of APD associated with cellulitis. 

## Case report 

We report the case of a 58-year-old Caucasian male with a previous medical history of maintenance hemodialysis for 5 years due to end-stage renal disease (ESRD) secondary to a histologically confirmed diabetic nephropathy. The patient had a diagnosis of type 2 diabetes requiring insulin, with 30 years of evolution and several subsequent microvascular and macrovascular complications, including retinopathy (treated with laser photocoagulation), ischemic heart disease (with three episodes of acute myocardial infarction), nephropathy, and peripheral artery disease (PAD; which led to bilateral lower limb amputation). Other antecedents included hypertension, obesity, a previous history of smoking, and a diagnosis of major depression. Usual medication included anti-platelet with acetylsalicylic acid and clopidogrel, apixaban, pantoprazole, amiodarone, losartan, atorvastatin, sevelamer, insulin, and escitalopram. Only apixaban was introduced in the 45 days prior to the consultation. Despite regular dialysis treatments, and without evidence of major intradialytic complications, an adequate kt/V was not reached. An angiography of the vascular access – a brachiobasilic arteriovenous fistula (AVF) on the left arm – was performed, showing no alterations. 

**Figure 1:**
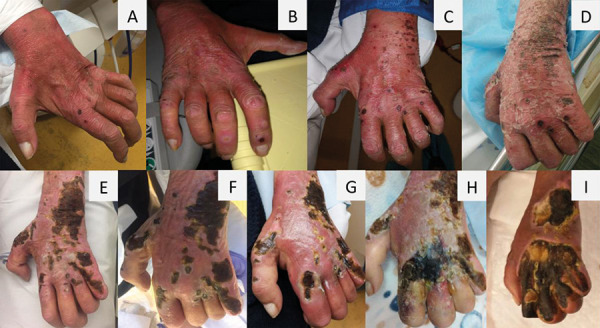
A and B: Aspect at presentation (D1). Umbilicated papules and some punctiform necrotic lesions. C: Larger and coalescent necrotic injuries (D19). D: First hospital admission; intense left-hand skin peeling (D22). E: Worst moment during hospitalization with subsequent modest improvements (no photographic record due to discharge against medical advice) (D32). F: Infectious intercurrence with cellulitis, first treated with vancomycin and gentamicin (D85). G: Cellulitis with poor response to previous antibiotic therapy, so flucloxacillin was initiated (D95). H: Extensive tissue necrosis of the dorsum of the left hand and associated bullous lesions (D113). I: Extensive area of tendon exposure and devitalized tissues, as observed the day before the amputation (D133).

Day 1: The patient presented with a 1-week history of painful, pruritic, umbilicated papules and some punctiform necrotic lesions in the left forearm and hand, right hand, and both amputation stumps (Figure 1A, B). Radial pulses and extremity temperature were normal, although the patient presented with pale fingers and prolonged capillary refill time. This patient presented with severe PAD; furthermore, a recent angiography of the vascular access was normal. 

Day 19: Despite a short initial course of topical corticosteroid, the lesions – after a short desquamative phase – evolved into larger and coalescent necrotic injuries (Figure 1C). 

In this context, three primary diagnostic hypotheses were considered: calciphylaxis, even though there was no dysregulated calcium-phosphorous metabolism or warfarin therapy present; vasculitis; and APD, given the visual aspect, severe pain, pruritis, and past medical history. 

**Figure 2:**
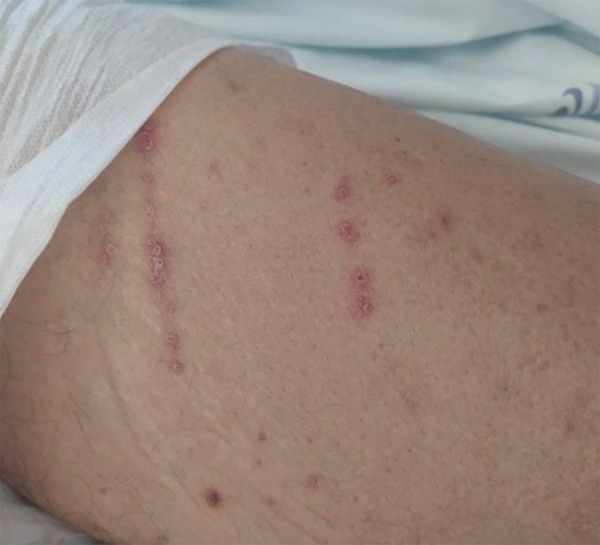
Positive Koebner phenomenon (appearance of new skin lesions at sites of minor trauma in otherwise healthy skin).

Day 22: The patient rapidly progressed and reported aggravated pain, while intense left-hand skin peeling and appearance (Figure 1D) of similar lesions on the trunk were evident. The patient was admitted to the hospital 3 weeks after initial presentation. The case was readily discussed with Dermatology specialists, who agreed with the hypothesis of APD. A skin biopsy of a trunk lesion was performed; while waiting for the result, treatment with topical sodium fusidate, betamethasone, and intradialytic sodium thiosulfate were initiated, with partial results. Histopathology findings were compatible with APD, while also excluding findings suggestive of calciphylaxis or vasculitis. The Koebner phenomenon was also evident (i.e., the appearance of new skin lesions at sites of minor trauma in otherwise healthy skin (Figure 2)). 

**Figure 3:**
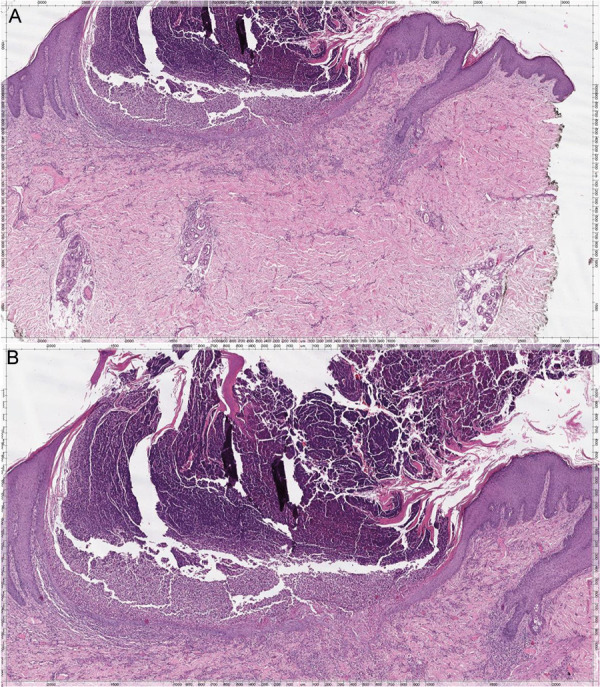
A: Microscopic findings of central plug with parakeratotic debris, degenerate collagen, and inflammatory cells. The epidermis deep to the plug is thinned and traversed focally by vertically oriented collagen Fibers (H & E, × 200). B: Closer view of the central plug (H & E, × 400).

The histology findings of the skin biopsy revealed a central plug with parakeratotic debris and degenerate collagen, associated with inflammatory cells. The epidermis deep to the plug was thinned and traversed focally by vertically oriented collagen fibers (Figure 3). These findings are consistent with APD. 

Although CKD and diabetes mellitus are clearly associated with APD, other potential causes were excluded – namely HIV, tuberculosis, hypothyroidism, liver disease, and hyperparathyroidism. 

Complications experienced in response to the skin biopsy procedure, including minor hemorrhage and inflammatory signs without evidence of infection, required a prolonged hospital stay until the patient requested to be discharged against medical advice on day 26 of hospitalization. The patient presented with modest improvements on implementation of topical therapy. Outpatient follow-up was requested with the Dermatology clinic upon discharge. Wound care was performed at community health center and at the dialysis center. 

Day 85: The patient maintained regular dialysis treatments at the dialysis center, but at 1.5 months later, he presented with left-hand erythema, edema, and warmth, suggestive of cellulitis. Vancomycin and gentamicin were empirically prescribed with slow response (Figure 1F); as such, flucloxacillin was initiated (Figure 1G, day 95). 

Day 113: Despite this, the patient presented to the emergency room with extensive tissue necrosis of the dorsum of the left hand and associated phlegmon/bullous lesions (Figure 1H). Computed tomography (CT) scan showed possible osteomyelitis, thickening, and densification of much of the subcutaneous tissue of the left hand and forearm, as well as important vascular calcifications at the radial and cubital levels. Antibiotic therapy with piperacillin-tazobactam was initiated at the hospital (adjusted based on renal function) and Vascular Surgery was contacted. The surgical department assumed access steal syndrome, as the vascular access was ipsilateral and the other lesions had evolved well, so an AVF ligation was performed, as was debridement of the phlegmon. 

Day 133: The patient evolved unfavorably with an extensive area of tendon exposure and devitalized tissues in the forearm, acute ischemia of the left index finger, difficult-to-treat cellulitis of the left hand, and severe pain (Figure 1I). Medical therapy was attempted by Plastic Surgery, which included topical therapy with povidone iodine for skin disinfection and wet compresses with Dakin solution, as well as maintenance of the antibiotic, with no success. Given the severity of the injuries and the patient’s comorbidity, a reconstructive solution was disregarded. The case was discussed with Orthopedic Surgery, where it was concluded that amputation was the most viable solution. The patient agreed, in a conscious and informed way, with the amputation of the left hand and forearm. During the procedure, purulent drainage from the extensor tendons of the patient’s fingers was sent for microbial culture, where it was revealed that *Serratia marcescens*, which is sensitive to gentamicin, was the causative organism. Blood cultures were negative. 

## Discussion 

APD pathogenesis is not completely clear, as some authors suggest that mechanical trauma from scratching and microvasculopathy associated with both diabetes and CKD are triggers, inducing collagen necrosis and the subsequent elimination [2, 4, 8]. Although this condition is self-limited, it is recurrent [7]. 

While visual examination can be suggestive of APD, histologic analysis is necessary to confirm its diagnosis. Clinically, APD lesions might appear as hyperpigmented or erythematous papules or nodules, occasionally umbilicated, and with a keratotic plug or central crust that is severely pruritic and, eventually, painful [1, 7]. These lesions regress within 6 – 8 weeks, leaving a keratotic cover, can easily bleed if removed, later resulting in scarring or hyperpigmentation [7]. These lesions are frequently seen in multiple locations, most frequently on the extensor surface of the extremities (limbs and hands) and trunk [2, 8]. Positive Koebner phenomenon is an important feature [2, 3, 10]. 

APD might be an underdiagnosed problem, as it is easily confused with other entities. Its differential diagnoses are extensive and include arthropod bites, folliculitis, prurigo nodularis, prurigo simplex, psoriasis, and lichen planus, if Koebner phenomenon is present, as well as calciphylaxis, among others [1]. 

Beyond histopathology, the difference between APD and calciphylaxis lies in the presence of pruritic papules or nodules, which can be found in APD, whereas calciphylaxis usually starts as violaceous mottling, progressing to painful plaques or nodules, and finally to necrotic ulcers [10]. As stated earlier, APD lesions are frequently seen in multiple locations, most frequently on the dorsum of the extremities (limbs and hands), trunk, and head [2, 8]. Calciphylaxis is often seen in the lower extremities, abdomen, and buttocks, related to the presence of large amounts of subcutaneous fat [10]. 

Treatment of APD can be particularly challenging, as lesions can persist or relapse, and chronic scars can develop [10]. Besides controlling the underlying disease, there are several treatment options available, but they are supported by weak scientific evidence, such as topical, systemic, or intralesional corticosteroids, topical keratolytics, antihistaminic and menthol-based lotions aimed to treat pruritus as well as topical and systemic retinoids, phototherapy, and antibiotics, among others [1, 3, 4, 8]. Hari Kumar et al. [13] reported on the data of 8 hemodialysis patients with diabetic kidney disease who presented with APD, all of whom showed significant resolution with topical glucocorticoid therapy. Further, renal transplantation has been shown to resolve hemodialysis-associated APD [4, 9]. 

A PubMed search was conducted by the authors in May 2020 with the terms “perforating dermatosis” + cellulitis and “acquired perforating dermatosis” + cellulitis with no results. To our knowledge, there are no previously described cases of ADP-related cellulitis. In this case scenario, we believe that the patient presented with an ADP relapse soon after discharge, which served as the entry point for the cellulitis to develop, and which was associated with the underlying severe vasculopathy due to type 2 diabetes and ESRD. By this point, the ADP progressed to a critical point of no return, resulting in ischemia and amputation. 

Although *Serratia* skin and soft tissue infections are uncommon, they mostly affect immunocompromised patients, namely those with renal failure and who are subjected to instrumentation and prior antibiotic therapy [11, 12]. Chronic vascular disease and venous insufficiency also predispose these patients to local infection. These cases are frequently resistant to conventional antibiotics, and surgery may be considered [11, 12]. Several presentations have been described, which include plaques, bullous cellulitis, nodules, or necrotizing cellulitis, and may lead to life-threatening sepsis if spread [12]. Awareness of this difficult-to-treat cellulitis that affects immunocompromised patients is essential to achieving better outcomes. 

## Conclusion 

APD can be associated with cellulitis, as well as its complications in patients with underlying severe vasculopathy. Awareness of this complication in cases of APD (especially if due to *Serratia*), along with timely referral and aggressive treatment, might improve the outcomes and quality of life of such patients. Histology is essential in determining the initial differential diagnosis, as APD might be an under- and misdiagnosed pathology. 

## Ethical considerations 

The authors confirm that there are no ethical issues to report in this manuscript or as part of its submission (including plagiarism, data fabrication, double publication). The patient provided his informed consent to be published as a case report. 

## Acknowledgment 

English-language editing of this manuscript was provided by Journal Prep Services. 

## Funding 

None. 

## Conflict of interest 

The authors declare no competing interests.
